# Relationship of Vitamin D Deficiency and Fatty Liver in Children as Defined by Multiple Imaging and Histologic Endpoints

**DOI:** 10.1097/PG9.0000000000000077

**Published:** 2021-04-30

**Authors:** Bryan Rudolph, Tyler Selig, Yingjie Li, Nadia Ovchinsky, Debora Kogan-Liberman, Mark C. Liszewski, Terry L. Levin, Michelle Ewart, Qiang Liu, Shankar Viswanathan, Juan Lin, Xiaonan Xue, Robert D. Burk, Howard D. Strickler

**Affiliations:** From the * Division of Pediatric Gastroenterology, Hepatology and Nutrition, Children’s Hospital at Montefiore, Bronx, NY; † Albert Einstein College of Medicine, Bronx, NY; ‡ Division of Pediatric Radiology, Children’s Hospital at Montefiore, Bronx, NY; § Division of Pediatric Pathology, Children’s Hospital at Montefiore, Bronx, NY; ∥ Department of Epidemiology and Population Health, Albert Einstein College of Medicine, Bronx, NY.

**Keywords:** nonalcoholic steatohepatitis, children, vitamin D, elastography

## Abstract

The relationship between vitamin D deficiency (VDD) and pediatric nonalcoholic fatty liver disease (NAFLD) remains uncertain due to conflicting results and few studies with histologic endpoints. We therefore used multiple imaging and histologic NAFLD endpoints to more comprehensively assess the association between VDD and NAFLD in a large pediatric population.

**Methods:**

Data were obtained from an ongoing pediatric NAFLD study in Bronx, NY. Briefly, overweight and obese children aged 2 to 18 years with alanine aminotransferase levels ≥35 U/L were serially enrolled. Liver biopsy was obtained in accordance with clinical guidelines. All participants had liver imaging, namely, controlled attenuation parameter to assess steatosis and, to assess fibrosis, vibration controlled transient elastography (FibroScan), and acoustic radiation force impulse imaging. Levels of 25-hydroxyvitamin D were measured serologically.

**Results:**

N = 276 (88%) of 315 participants had 25-OH vitamin D results, of whom 241 (87%) were Hispanic, 199 (72%) were male, and 92 (33%) underwent liver biopsy. VDD was univariately associated with high waist circumference (*P* = 0.004), high-density lipoprotein level (*P* = 0.01), season (*P* = 0.009), and controlled attenuation parameter score (*P* = 0.01). In multivariate analysis, only waist circumference (*P* = .0002) and biopsy inflammation grade (*P* = 0.03) were associated with VDD, though the latter had not approximated statistical significance in univariate analysis (*P* = 0.56). There was no association between VDD and hepatic steatosis, ballooning, NAFLD activity score, and acoustic radiation force impulse or vibration controlled transient elastography elasticity scores.

**Conclusions:**

VDD was not associated with NAFLD defined by imaging and histologic endpoints, except for a possible relation with histologic inflammation grade.

**What Is Known**

- Vitamin D deficiency and its relationship with pediatric nonalcoholic fatty liver disease (NAFLD) remain uncertain due to limited and conflicting prior data.

**What Is New**

- This methodologically rigorous study utilized imaging and histologic endpoints to more comprehensively assess the relationship between vitamin D deficiency and pediatric NAFLD.
- Most prior studies did not control for adiposity (eg, waist circumference), which is strongly associated with vitamin D deficiency. After statistical adjustment for waist circumference in this study, the univariate association between vitamin D deficiency and CAP (ie, steatosis) was no longer statistically significant.
- VCTE, CAP, and ARFI are not associated with vitamin D deficiency among children with NAFLD.

## INTRODUCTION

Nonalcoholic fatty liver disease (NAFLD) is strongly associated with obesity and encompasses 2 distinct conditions: nonalcoholic fatty liver (NAFL) and nonalcoholic steatohepatitis (NASH). Both are characterized by the presence of steatosis, whereas NASH is defined by the additional features of inflammation and hepatocyte ballooning. Over time, patients with NAFLD may develop fibrosis and cirrhosis.

Vitamin D deficiency (VDD) has long been suspected to play a role in NAFLD development and progression. Animal data, for example, have shown that fibrosis develops in vitamin D receptor knockout mice but is attenuated after pharmacological receptor activation in CCl_4_-treated animals.^1^ Laboratory studies have also shown that vitamin D supplementation reduces the expression of profibrotic mediators such as platelet-derived growth factor and transforming growth factor β, which suppress hepatic stellate cell proliferation and extracellular matrix deposition.^1–3^

However, human data regarding VDD and its relation with NAFL and NASH, especially in children, are limited and conflicting.^4–14^ Among the few pediatric studies that use histologic data, 2 European groups found an association between VDD and NAFLD Activity Score (NAS), though only one of these studies also found an association with NASH and fibrosis.^11,12^ Conversely, a multicenter study in the United States found no relationship between VDD and histological NAFLD severity.^13^ Another large, single-center US study paradoxically found an association between vitamin D *insufficiency,* but not VDD, with hepatic fibrosis and NAFLD.^14^ Most prior studies did not control for adiposity, which is strongly correlated with NAFLD and inversely correlated with vitamin D levels.^15–17^ We therefore sought to more comprehensively study the relationship between VDD and NAFLD using both histologic and noninvasive (ie, elastography) endpoints while controlling for multiple potential confounders, including adiposity. Given the lack of pediatric thresholds for elastography, data were analyzed in multiple ways.

## METHODS

### Design Overview

Data were obtained from a large, ongoing study of pediatric NAFLD. The methodology for this study, including a flow diagram and a detailed protocol, has been previously published.^18^ In brief, asymptomatic children between 2 and 18 years of age with a body mass index (BMI) ≥85th percentile for age and an elevated alanine aminotransferase (ALT) (≥35 U/L) were serially enrolled through an NAFLD specialty clinic at the Children’s Hospital at Montefiore in the Bronx, New York. While lower pediatric thresholds are often utilized, ALT thresholds in this study were determined a priori with input from the institutional review board, so that biopsies or research tests not be performed in children with borderline ALT elevation.^18^ Alternative liver disease such as autoimmune and viral hepatitis, Wilson’s disease, celiac disease, thyroid or muscle disorders were ruled out via standard laboratory testing. Clinical and anthropometric data, elastography, and serum specimens were obtained at enrollment and at 6-month intervals. Liver biopsy was performed as clinically indicated. All patients or their parents/guardians gave their written consent or assent for participation. The study was approved by our Institutional Review Board and follows all applicable rules for data collection and privacy.

### Liver Biopsy

Patients with persistent ALT elevation lasting more than 6 months were offered a liver biopsy per professional society guidelines.^19^ Histologic specimens were de-identified and interpreted according to NASH Clinical Research Network guidelines by a panel comprised of two blinded, expert pathologists. Histologic variables included steatosis, inflammation, ballooning, NAS, and fibrosis.

### Vitamin D

At baseline, 25-hydroxyvitamin D (25(OH)D) assays were performed using a competitive immunoassay (Roche Diagnostics, Indianapolis, IN) at a CLIA-certified centralized institutional laboratory. For purposes of analysis, 25(OH)D levels were categorized a priori as deficient, insufficient, or sufficient if the level was ≤20, 20 to 30, or ≥30 ng/mL, respectively. Because serum 25(OH)D levels fluctuate with sunlight exposure, the season in which the measurement was taken—a potential confounder—was also recorded.

### Imaging Tests

Two forms of elastography, vibration controlled transient elastography (VCTE, FibroScan, Echosens, France) and acoustic radiation force impulse (ARFI, Philips, Netherlands) imaging, were performed at enrollment and previously described intervals, including at the time of biopsy.^18^ Elastography is a noninvasive imaging modality and an indirect measure of liver fibrosis. The test is performed by placing a transducer on the patient’s skin overlying the liver and applying a nonpainful force. The force generates a shear wave within the liver and, by measuring the velocity of that shear wave, tissue elasticity (ie, fibrosis) can be measured. Elasticity forms vary based on the methods used to generate the force and measure shear wave velocity but are generally comparable in efficacy.^20^ VCTE can measure both fibrosis and shear wave diffusion within the liver to calculate a controlled attenuation parameter (CAP) score, an indirect measure of hepatic steatosis.

Given the paucity of prior data in children, several ways of parameterizing these data were examined, including continuous, ordinal, and binary. VCTE, for example, was dichotomized into groups with values <4.9 or ≥4.9 kPA. This threshold was chosen because it has been shown to distinguish between the absence (F0) and presence of any (≥F1) fibrosis in adults and fit the current data.^21^ Similarly, CAP values were stratified according to those with a score <280 or ≥280 dB/m, which corresponds to adult patients with grades 0-1 versus 2-3 steatosis, respectively.^21^ ARFI values were dichotomized by those with values <1.3 or ≥1.3 m/s, which corresponds to adult patients with significant (≥F2) fibrosis.^22^ All stratification decisions were made a priori.

### Statistical Analysis

In preliminary data analysis, descriptive statistics were estimated for demographic, metabolic, imaging, and biopsy variables. Contingency tables were constructed for frequency data, and mean, median, interquartile range, and assessment of normality for continuous variables. Spearman rank correlations were measured as an initial, univariate estimate of association between covariates, anthropometric variables, and 25(OH)D. Chi-square test for binary data, Chi-square and the Mantel extension test for linear trends for ordinal data were used to assess differences in these values between groups. Given the gender distribution of our cohort, bivariate testing was also performed in a male-only cohort. These analyses were performed with 25(OH)D expressed categorically (deficient, insufficient, and sufficient) as well as a binary variable (deficient or not deficient). Because relatively few patients were vitamin D sufficient, only binary outcomes were reported.

Multivariate logistic regression models were then used to examine the relationship between dichotomized imaging variables, biopsy variables, and 25(OH)D level while controlling for age, gender, Hispanic ethnicity, waist circumference, high-density lipoprotein (HDL), homeostatic model assessment of insulin resistance, and season. Estimates of odd ratio along with its 95% confidence interval were reported. Two-sided *P* values <0.05 were considered statistically significant. To exclude the possibility that use of vitamin D replacement therapy within 90 days of biopsy may have affected the findings, in sensitivity analysis we recharacterized patients on vitamin D replacement therapy within 90 days of biopsy as 25(OH)D sufficient, regardless of baseline level. All testing was performed using SAS (version 9.4).

## RESULTS

At the time of analysis, 315 subjects were enrolled in this ongoing study. Of these, 276 children (88%) had recorded 25(OH)D levels and 92 (33%) underwent a liver biopsy. VCTE and CAP were performed in all patients and ARFI in 259 (94%). Among the 39 participants without vitamin D data, 11 subjects (28%) had been lost to follow up, 12 (30%) had missing data, and 16 (41%) were pending blood draw.

The median (interquartile range) age at enrollment was 13.1 (10.8 to 16.1) years, 199 (72%) were male, and 241 (87%) were Hispanic (primarily of Mexican descent). The median ALT and BMI z-score was 55 U/L (38 to 90) and 2.25 (1.90 to 2.46), respectively. Twenty-eight children (10.1%) were overweight and the remainder were obese (Table 1).

**TABLE 1.** Demographic characteristics of participants with NAFLD

Variable	Result
Age (y)	13.1 (10.8–16.1)
Sex (male)	199 (72.1%)
Ethnicity (Hispanic)	241 (87.3%)
BMI (kg/m^2^)	30.4 (27.1–34.6)
BMI Z-score	2.25 (1.90–2.46)
Overweight	28 (10.1%)
Obese	248 (89.9%)
Waist circumference (cm), N = 298	100.7 (90.5–112.3)
GGT (U/L), N = 314	28 (21–44)
ALT (U/L)	55 (38–90)
AST (U/L)	35 (26–49)
Total cholesterol (mmol/L), N = 268	158 (139–182)
Triglyceride (mmol/L), N = 259	131 (90–178)
HDL (mmol/L), N = 268	39 (34–47)
LDL (mmol/L), N = 268	87 (72–111)
Fasting glucose (mg/dL), N = 272	88 (83–95)
Fasting insulin (uIU/mL), N = 246	23.5 (15.6–38.9)
HbA_1_c, N = 274	5.4 (5.2–5.7)
HOMA-IR, N = 244	5.4 (3.4–8.2)
25(OH)D (ng/mL)	18.4 (14.7–22.8)
25(OH)D deficient	169 (62%)
25(OH)D insufficient	92 (33.3%)
25(OH)D sufficient	15 (5.4%)
Season, N = 266	
Winter	65 (24.4)
Spring	56 (21.1)
Summer	52 (19.6)
Fall	93 (35)
Imaging	
VCTE (kPa)	5.2 (4.4–6.3)
CAP (dB/m)	307 (279–341)
ARFI (m/s), N = 259	1.23 (1.16–1.34)
Biopsy, N = 92	
Steatosis grade ≥ 2	47 (51.1%)
Inflammation grade ≥ 2	32 (34.8%)
Ballooning grade ≥ 1	55 (59.8%)
NAS	5 (4-6)
Fibrosis grade ≥ 1	53 (57.6%)

Continuous and categorical data are presented as median (interquartile range) or number (percentage), respectively. Data reported is for 276 patients unless otherwise specified. 25(OH)D = 25-hydroxyvitamin D; ALT = alanine aminotransferase; ARFI = acoustic radiation force impulse; AST = aspartate aminotransferase; BMI = body mass index; CAP = controlled attenuation parameter; GGT = γ-glutamyl transferase; HbA1c = hemoglobin A1c; HDL = high-density lipoprotein; HOMA IR = Homeostatic Model Assessment of Insulin Resistance; LDL = low-density lipoprotein; NAFLD, nonalcoholic fatty liver disease; NAS, NAFLD activity score; VCTE = vibration controlled transient elastography.

The median 25(OH)D level at baseline was 18.4 (14.7 to 22.8) ng/mL; 169 (62%) were vitamin D deficient, 92 (33%) were insufficient, and 15 (5.4%) were sufficient. The median time between 25(OH)D levels and liver biopsy was 31.5 days (12 to 68) and 16 patients were prescribed vitamin D replacement therapy within 90 days of biopsy. The median time between 25 (OH)D and VCTE, CAP, and ARFI were 6, 6, and 147 days, respectively.

Levels of 25(OH)D had modest but statistically significant Spearman correlations with waist circumference (r −0.177, *P* = 0.004) and HDL (r 0.156, *P* = 0.01) as well as univariate associations with seasonality (*P* = 0.009) and CAP score (*P* = 0.01) (Tables 2 and 3). These findings did not meaningfully change when analysis was limited to males. Furthermore, among males only, there was a statistically significant correlation between 25(OH)D and triglyceride level (−0.187, *P* = 0.01). However, there were no associations/correlations between the 25(OH)D level and other covariates, nor with categorically expressed outcome variables such as VCTE (*P* = 0.16), ARFI (*P* = 0.29), histologic steatosis grade (*P* = 0.96), ballooning (*P* = 0.39), NAS (*P* = 0.85), or fibrosis (*P* = 0.61) (see Table 3). Results were unchanged when outcome variables such as elastography were expressed continuously (data not shown). Analysis of 25(OH)D as a continuous variable also did not affect these findings nor did recharacterizing the 16 patients using vitamin D replacement as having sufficient 25(OH)D levels (data not shown).

**TABLE 2.** Correlation of 25(OH)D with anthropometric and laboratory values

	All participants (N = 276)	*P*	Male only (N = 199)	*P*
Age (y)	−0.104	0.09	−0.104	0.14
BMI Z-score	−0.023	0.70	−0.086	0.23
Waist circumference (cm)	−0.177	**0.004**	−0.179	**0.01**
GGT (U/L)	−0.068	0.26	−0.076	0.29
ALT (U/L)	−0.087	0.15	−0.074	0.29
AST (U/L)	−0.078	0.19	−0.106	0.14
Total cholesterol (mmol/L)	0.030	0.63	−0.024	0.73
Triglyceride (mmol/L)	−0.089	0.15	−0.187	**0.01**
HDL (mmol/L)	0.156	**0.01**	0.193	**0.007**
LDL (mmol/L)	0.034	0.57	−0.014	0.85
Fasting Glucose (mg/dL)	−0.073	0.23	−0.137	0.06
Fasting Insulin (uIU/mL)	−0.113	0.08	−0.090	0.24
HbA_1_c (%)	−0.070	0.25	−0.091	0.20
HOMA IR	−0.108	0.09	−0.104	0.17

All variables were characterized continuously and analyzed via Spearman correlation [r (*P* value)]. *P* values <0.0.5 were considered significant in bold. 25(OH)D = 25-hydroxyvitamin ALT = alanine aminotransferase; AST = aspartate aminotransferase; BMI = body mass index; GGT = γ-glutamyl transferase; HbA_1_c = hemoglobin A1c; HDL = high-density lipoprotein; HOMA IR = Homeostatic Model Assessment of Insulin Resistance; LDL = low-density lipoprotein.

**TABLE 3.** Association of 25(OH)D with categorical variables and categorized outcomes

	All participants (N = 276)			Male only (N = 199)		
	Deficient	Not deficient	*P*	Deficient	Not deficient	*P*
Sex			0.61			—
Male	120 (43.5%)	79 (28.6%)		—	—	
Female	49 (17.8%)	28 (10.1%)		—	—	
Ethnicity			0.83			0.30
Hispanic	147 (53.3%)	94 (34.1%)		100 (50.3%)	70 (35.2%)	
Non-Hispanic	22 (8.0%)	13 (4.7%)		20 (10%)	9 (4.5%)	
Season			**0.009**			**0.02**
Spring	41 (15.4%)	15 (5.6%)		31 (16.1%)	9 (4.7%)	
Summer	31 (11.7%)	21 (7.9%)		23 (12%)	14 (7.3%)	
Fall	49 (18.4%)	44 (16.5%)		33 (17.2%)	36 (18.8)	
Winter	42 (15.8%)	23 (8.6%)		29 (15.1%)	17 (8.9%)	
VCTE (kPa)			0.16			0.49
< 4.9	58 (21.1%)	46 (16.7%)		41 (20.7%)	31 (15.7%)	
≥ 4.9	110 (40%)	61 (22.2%0		78 (39.4%)	48 (24.2%)	
CAP score (dB/m)			**0.01**			0.39
< 280	34 (12.4%)	36 (13.1%)		24 (12.1%)	20 (10.1%)	
≥ 280	134 (48.7%)	71 (25.8%)		95 (48%)	59 (29.8%)	
ARFI (m/s)			0.29			0.36
<1.3	101 (39%)	71 (27.4%)		64 (34.8%)	47 (25.5%)	
≥1.3	57 (22%)	30 (11.6%)		47 (25.5%)	26 (14.1%)	
Steatosis			0.96			0.90
≤2	28 (30.4%)	17 (18.5%)		20 (29.4%)	12 (17.6%)	
3	29 (31.5%)	18 (19.6%)		23 (33.8%)	13 (19.1%)	
Inflammation			0.59			0.56
<2	36 (39.1%)	24 (26%)		28 (41.2%)	18 (26.5%)	
≥2	21 (22.8 %)	11 (12%)		15 (22.1%)	7 (10.3%)	
Ballooning			0.39			0.14
0	21 (22.8%)	16 (17.3%)		13 (19.1%)	12 (17.6%)	
≥ 1	36 (39.1%)	19 (20.7%)		30 (44.1%)	13 (19.1%)	
NAS			0.85			0.30
0–3	13 (14.1%)	9 (9.8%)		7 (10.3%)	7 (10.3%)	
4–5	28 (30.4%)	18 (19.6%)		23 (33.8%)	14 (20.6%)	
≥ 6	16 (17.4%)	8 (8.7%)		13 (19.1%)	4 (5.9%)	
Fibrosis grade			0.61			0.67
0	23 (25%)	16 (17.4%)		15 (22.1%)	10 (14.7%)	
≥ 1	34 (37%)	19 (20.6%)		28 (41.1%)	15 (22.1%)	

All variables were categorized categorically and analyzed via Chi-squared testing. *P* values <0.0.5 were considered significant in bold. 25(OH)D = 25-hydroxyvitamin D; ARFI = acoustic radiation force impulse; CAP = controlled attenuation parameter; VCTE = vibration controlled transient elastography.

The association between 25(OH)D and CAP was no longer statistically significant in multivariate logistic regression models (OR 1.0, 95% confidence interval 0.95-1.05, *P* = 0.87) when controlling for waist circumference, with or without other covariates such as age, gender, Hispanic ethnicity, HDL, homeostatic model assessment of insulin resistance, and season. Conversely, 25(OH)D levels were associated with inflammation score in these same multivariate models (OR 0.89, 95% confidence interval 0.81-0.99, *P* = 0.03), though this association was not initially statistically significant in univariate analysis (*P* = 0.56) (see Tables 3 and 4).

**TABLE 4.** Logistic regression for CAP and inflammation

	CAP		Inflammation	
	OR (95% CI)	*P*	OR (95%CI)	*P*
Waist circumference	1.08 (1.04, 1.12)	**0.0002**	0.99 (0.94, 1.05)	0.73
Age	0.94 (0.82, 1.09)	0.42	1.14 (0.90, 1.46)	0.27
Male gender	1.24 (0.57, 2.68)	0.59	0.40 (0.11, 1.47)	0.17
Hispanic ethnicity	2.26 (0.77, 6.64)	0.14	2.94 (0.25, 34.29)	0.39
Vitamin D	1.0 (0.95, 1.05)	0.87	0.89 (0.81, 0.99)	**0.03**
HDL	0.99 (0.96, 1.03)	0.71	1.06 (0.996, 1.14)	0.07
HOMA-IR	1.06 (0.97, 1.16)	0.17	0.962 (0.89, 1.05)	0.39
Season		0.85		0.09
Spring vs winter	1.30 (0.44, 3.84)		0.18 (0.03, 0.94)	
Summer vs winter	0.88 (0.31, 2.53)		0.96 (0.18, 5.08)	
Fall vs winter	0.86 (0.33, 2.24)		1.88 (0.48, 7.45)	

Logistic regression for CAP and inflammation grade with exposure variables expressed continuously, unless otherwise specified. Results provided as OR with 95% CI. *P* values <0.0.5 were considered significant in bold. CAP = controlled attenuation parameter; CI = confidence interval; HDL = high-density lipoprotein; HOMA IR = Homeostatic Model Assessment of Insulin Resistance; OR = odds ratio.

## DISCUSSION

Experimental data suggest that vitamin D plays a role NAFLD pathogenesis, but epidemiologic data are decidedly more mixed. As discussed, pediatric data on the association between VDD and NAFLD are limited and conflicted. Results from adult studies are similarly conflicted^23–31^ (see Table 5). In fact, even published meta-analyses have reached different conclusions.^32–34^

**TABLE 5.** Selected studies on correlation of 25(OH)D to NAFLD

Study	Population	Country	Sample size	Outcome	Result
Cho et al^4^	Adolescents	Korea	3,878	ALT	Vitamin D associated with NAFLD with OR of 1.77
Mohamed et al^5^	Children	Egypt	47	Ultrasound	Lower vitamin D levels associated with NAFLD
Sezer et al^6^	Children	Turkey	111	Ultrasound	No association between VDD and NAFLD
Chang et al^7^	Children	Korea	94	Ultrasound/ALT	No association between vitamin D and NAFLD
Malespin et al^8^	Children	USA	407	ALT	Vitamin D levels associated with NAFLD with OR 0.83
Yildiz et al^9^	Children	Turkey	101	Ultrasound	Lower vitamin D levels associated with NAFLD
Black et al^10^	Adolescents	Australia	994	Ultrasound	Vitamin D levels associated with NAFLD with OR 0.74
Nobili et al^11^	Children	Italy	74	Biopsy	Lower vitamin D associated with NASH, stage 1, 2 fibrosis
Hourigan et al^13^	Children	USA	102	Biopsy	No association between vitamin D and NAFLD
Yodoshi et al^14^	Children	USA	234	Biopsy	Vitamin D insufficiency associated with fibrosis
Targer et al^23^	Adults	Italy	60	Biopsy	Vitamin D associated with steatosis, inflammation, fibrosis
Eraslan et al^25^	Adults	Turkey	63	Biopsy	Vitamin D correlated with NAS with r −0.317
Dasarathy et al^26^	Adults	USA	148	Biopsy	Lower vitamin D levels associated with ballooning
Diez Rodriguez et al^27^	Adults	Spain	110	Biopsy	No association between vitamin D and NAFLD
Anty et al^28^	Adults	France	398	Biopsy	No association between vitamin D and NAFLD
Nelson et al^29^	Adults	USA	190	Biopsy	VDD associated with NASH (OR 3.15), fibrosis (OR 2.38)
Luger et al^30^	Adults	Austria	46	Biopsy	Lower vitamin D levels associated with fibrosis
Patel et al^31^	Adults	USA	244	Biopsy	No association between vitamin D and NAFLD

Selected pediatric and adult studies on the association between vitamin D to NAFLD. ALT = alanine aminotransferase; NAFLD = nonalcoholic fatty liver disease; NAS = NAFLD activity score; NASH = nonalcoholic steatohepatitis; OR = odds ratio; VDD = vitamin D deficiency.

It is important to recognize, however, that these studies utilize multiple serum assays, definitions for VDD, diagnostic criteria for NAFLD, and patient cohorts. Furthermore, liver biopsy is an imperfect “gold standard” test and suffers from sampling, interobserver and intraobserver variability.^35–37^

Given the ease in which vitamin D levels can be drawn and replaced, the absence of other NAFLD pharmacotherapy, and the clinical focus providers place on vitamin D, clarifying this association is important. This study therefore used noninvasive imaging and histologic endpoints to carefully study the relationship of VDD with NAFLD in children. In our study, VDD was not associated with hepatic steatosis, fibrosis, NAS, ARFI, VCTE, or CAP. While there was a statistically significant association of VDD with histologic inflammation grade, it was of moderate strength and had not been observed in univariate analysis. We therefore cannot exclude the possibility that this was a spurious observation related to multiple statistical testing. However, in general, whether assessed by liver biopsy or elastography, VDD was not associated with NAFLD in children.

The noninvasive imaging tests used in this study are widely considered among the best noninvasive options for disease stratification currently available in pediatric patients.^20^ CAP, for example, has been shown to highly correlate with hepatic steatosis.^38^ Moreover, ARFI and VCTE have high predictive values for diagnosing hepatic fibrosis, with an area under the receiver operator curve (AUROC) approaching 0.85 and 0.91, respectively.^39,40^ Although there are no established pediatric thresholds for these imaging modalities, elastography is clinically important and validated in adults. Furthermore, elastography results were analyzed multiple ways (ie, as a continuous, ordinal, and/or binary variable). There was no statistically significant association with VDD in any of these analyses regardless of variable parameterization. This provides additional data, beyond analysis of biopsied patients, that there is no association between vitamin D and NAFLD within our cohort. In addition to a rigorous methodologic and statistical analysis, further strengths include the use of an expert pathology panel to interpret biopsy results and control for multiple confounders, including adiposity (ie, waist circumference).

This study also had several limitations. In particular, this study was cross-sectional in design and values such as 25(OH)D and waist circumference may vary over time. The findings may also not be generalizable to all pediatric populations. The participants in this study were predominantly Hispanic, a population with high susceptibility to liver disease and progression due to high carrier frequency of the I148M PNPLA3 polymorphism.^41^ Thus, the study is a representative of an important at-risk population but may not be fully representative of the general US population. Similarly, only 5.4% of our cohort were vitamin D sufficient, which may reflect our cohort’s race and ethnicity or poor access to healthy food. Though we addressed this statistically through comprehensive analysis (eg, characterizing 25(OH)D as a continuous and binary variable), this finding may also limit generalizability. Future studies should examine other populations within and beyond the United States.

## CONCLUSION

This study utilized biopsy and noninvasive imaging modalities to demonstrate no significant association between VDD and NAFLD other than a weak and uncertain association with histologic inflammation. Clinical trials of vitamin D may therefore be unnecessary unless new, promising, cross-sectional data emerge.

## ACKNOWLEDGMENTS

All authors made substantial contributions to the work, as outlined below; critically revised or drafted the work for critical content; approved the final draft; and agree to be accountable for all aspects of the work. All authors were involved in collection, analysis, and interpretation of the data and review and approval of the manuscript. B.R. and H.D.S. were involved in study design and concept. : B.R. was involved in drafting the manuscript.
